# Study of Arsenic Sulfide in Solid Tumor Cells Reveals Regulation of Nuclear Factors of Activated T-cells by PML and p53

**DOI:** 10.1038/srep19793

**Published:** 2016-01-22

**Authors:** Wenping Ding, Yingying Tong, Xiuli Zhang, Minggui Pan, Siyu Chen

**Affiliations:** 1 Department of Oncology, Xin Hua Hospital Affiliated to Shanghai Jiao Tong University School of Medicine, Shanghai, China; 2 Department of Oncology and Hematology, Kaiser Permanente Medical Center, Santa Clara, CA; 3 Kaiser Permanente Division of Research, Oakland, CA, USA

## Abstract

Arsenic sulfide (AS) has excellent cytotoxic activity in acute promyelocytic leukemia (APL) but its activity in solid tumors remains to be explored. Here we show that AS and cyclosporine A (CsA) exerted synergistic inhibitory effect on cell growth and c-Myc expression in HCT116 cells. AS inhibited the expression of PML, c-Myc, NFATc1, NFATc3, and NFATc4, while stimulating the expression of p53 and NFATc2. Knockdown of PML reduced NFATc1, NFATc2, NFATc3 and NFATc4 expression while overexpression of p53 stimulated NFATc2-luciferase activity that was further augmented by AS by binding to a set of p53 responsive elements (PREs) on the NFATc2 promoter. Additionally, overexpression of p53 suppressed NFATc3 and NFATc4. Reciprocally, NFATc3 knockdown enhanced p53 while reducing MDM2 expression indicating that NFATc3 is a negative regulator of p53 while a positive regulator of MDM2, consistent with its tumor-promoting property as knockdown of NFATc3 retarded cell growth *in vitro* and tumor growth in xenograft. In patients with colon cancer, tumor expression of NFATc2 correlated with superior survival, while nuclear NFATc1 with inferior survival. These results indicate that AS differentially regulates NFAT pathway through PML and p53 and reveal an intricate reciprocal regulatory relationship between NFAT proteins and p53 pathway.

Arsenic trioxide (As_2_O_3_, ATO) is a FDA approved drug that has dramatically improved the survival of patients with APL when combined with all-trans retinoid acid (ATRA)^1^,^2^. Arsenic sulfide (As_4_S_4,_ AS) has similarly excellent activities in APL^2^,^3^ but its activity in solid tumors remains to be explored. ATO showed modest cytotoxic activity in solid tumors^4^. Mechanistically in APL cells ATO directly binds to the cysteine residues in the zinc-finger domain of the RBCC domain of PML/RARα and PML and brings them to small ubiquitin-like modifier (SUMO)-conjugating enzyme UBC9 for SUMOylation followed by degradation, leading to APL cell differentiation^5^. In chronic myelogenous leukemia (CML) cells, AS inhibits self-ubiquitination of c-CBL by binding to its RING domain, hence enhancing the ability of c-CBL to degrade its target proteins including BCR-ABL^6^. AS and ATO induce ROS and regulate other signaling pathways including the downregulation of NF-κB, inhibition of JAK-STAT, JNK, MEK, Bax/BCL2 as well as stimulating p53 and autophagy^2^,^4^,^7^.

PML is considered a tumor suppressor gene and a p53 transcriptional cofactor for certain targets^8^. Most studies have shown an intricate and cooperative relationship between these two important proteins. PML is capable of protecting p53 stability by sequestering MDM2 to the nucleolus^9^. In fibroblasts, PML promotes p53 acetylation to induce senescence in response to Ras oncogenic signal^10^. PML contains p53 responsive elements (PREs) and is a direct target of p53 and potentiates the anti-proliferative effect of p53^11^. However, in the PML^−/−^ splenocytes, p53 expression was stimulated by arsenic to the same level as in the wild-type cells^12^, indicating PML is not essential for p53 expression.

Classic Nuclear Factor of Activated T-cells (NFAT) gene products are transcription factors consisting of NFATc1, NFATc2, NFATc3, and NFATc4, activated through dephosphorylation by serine/threonine phosphatase calcineurin to unmask their nuclear localization signal sequence leading to nuclear import^13^. CsA inhibits calcineurin-NFAT pathway by binding to calcineurin to block the dephosphorylation of NFAT proteins^13^,^14^,^15^. NFAT play critical roles in numerous biological functions including angiogenesis, cardiovascular development, immune regulation, bone homeostasis, etc.^13^,^14^,^15^. More recently studies have shown that NFAT can be involved in the malignancies^16^. Many studies have shown that NFAT proteins can promote angiogenesis, enhance invasion, and induce cell proliferation in malignant cells. For example, NFATc1 was found to be overexpressed in many pancreatic cancers and promote proliferation^17^,^18^. In CML cells NFATc1 conferred resistance to imatinib treatment by activating Wnt pathway^19^. NFATc2 was found to increase the invasiveness of breast cancer^20^. NFATc3 increased the aggressiveness of angiosarcoma^21^. However, other studies have also shown that NFATc2 and NFATc3 can induce cell arrest in some cancer cells acting as tumor suppressors^22^,^23^. These findings indicate NFAT family members play different roles in different cellular context in the regulation of cell growth and differentiation.

In this study we have uncovered a novel and intricate mechanism of NFAT gene expression regulation by PML and p53 pathways by exploring the cytotoxic effect of AS in solid tumor cells. We found that AS and CsA synergistically inhibited cell growth and c-Myc expression indicating that AS and CsA share similar targets. Indeed, AS inhibited NFATc1, NFATc3, NFATc4, as well as PML and c-Myc, but stimulated NFATc2 and p53 expression. Knockdown of PML reduced the expression of all four NFAT proteins and c-Myc while knockdown of p53 or p53 inhibition by a p53 inhibitor abrogated the ability of AS to stimulate NFATc2 expression. Overexpression of p53 repressed both NFATc3 and NFATc4. Reciprocally NFATc3 repressed p53 and stimulated MDM2. Knockdown of NFATc3 inhibited cell growth in *vitro* and tumor growth in xenograft. P53 utilizes a set of NFATc2 promoter binding sites (PREs) to maintain its basal level but switches to a different set of PREs to drive its expression upon AS stimulation indicating an elegant regulatory mechanism by p53. In patients with colon cancer, tumor expression of nuclear NFATc1 was associated with inferior survival while tumor expression of NFATc2 correlated with superior survival. Our results reveal a novel mechanism operated by arsenic through modulating a previously unknown yet complex relationship involving NFAT, PML and p53 pathways. These results also have important implications in the regulation of many physiologic and pathologic processes involving NFAT pathway.

## Results

### AS and CsA synergistically inhibited colon cancer cell growth

**Figure 1:**
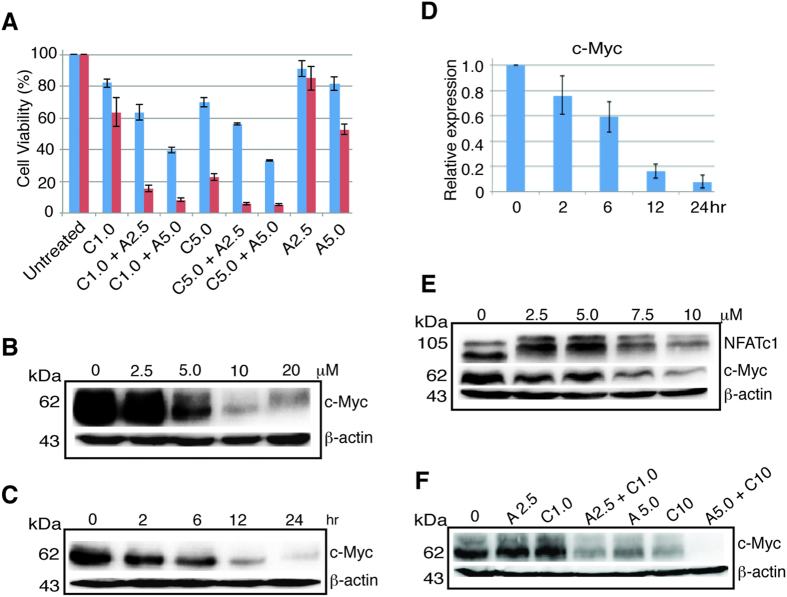
AS and CsA exert synergistic cell killing and c-Myc downregulation. (**A**) HCT116 cells were treated with CsA (C) or AS (A) alone or in combination in different concentrations as indicated in μM, for 24 (blue) or 48 (red) hours. Statistically significant difference was seen in all comparisons between cells treated with CsA or AS alone and cells treated with CsA plus AS in same concentration. Data represents one of the three identical experiments with similar results. Very significant statistical difference was detected for all four combinations versus the corresponding single agent treatment (see Table S1). (**B**) HCT116 cells were treated with AS in different concentration as indicated for 24 hours and blotted with antibody against c-Myc and β-actin. (**C**) HCT116 cells were treated with 10 μM AS for different time course (hour) as indicated and blotted with antibody against c-Myc and β-actin. (**D**) RT-PCR of c-Myc after HCT116 cells were treated with 10 μM AS from 2 to 24 hours. Error bar represents the standard deviation (SD) of the results of three separate experiments. (**E**) HCT116 cells were treated with CsA in different concentration as indicated for 24 hours and blotted with the antibody against NFATc1, c-Myc and β-actin. (**F**) HCT116 cells treated with AS or CsA alone or in combination for 24 hours, lysates were probed with antibody against c-Myc and β-actin. kDa, molecular weight.

We asked if AS and CsA in combination might have synergistic cell killing effect in colon cancer cells. We treated HCT116, a colon cancer cell line carrying wild-type p53 with different concentrations of AS and CsA either alone or in combination. At 1.0 and 5.0 μM CsA showed modest cell killing effect in both 24 and 48 hours (Fig. 1A, C1.0 and C5.0). At 5.0 μM AS showed modest killing at 48 hours (Fig. 1A, A5.0). However, when AS and CsA were combined, the killing effect was much more profound and synergistic compared to either agent alone in both 24 and 48 hours (Fig. 1A and Table S1). AS exerted potent inhibitory effect on c-Myc expression in a dose- and time- dependent manner (Fig. 1B,C). By RT-PCR AS also inhibited c-Myc expression in a time-dependent fashion (Fig. 1D). CsA showed modest inhibition on c-Myc at low concentration, but at 7.5 and 10 μM it showed potent effect (Fig. 1E). When CsA and AS were combined, the inhibition on c-Myc was synergistic in both low and high dose levels (Fig. 1F). These results suggested that AS and CsA affect a common signaling pathway and share similar targets.

### AS inhibited NFATc1, NFATc3, and NFATc4, but stimulated NFATc2 expression transcriptionally

**Figure 2:**
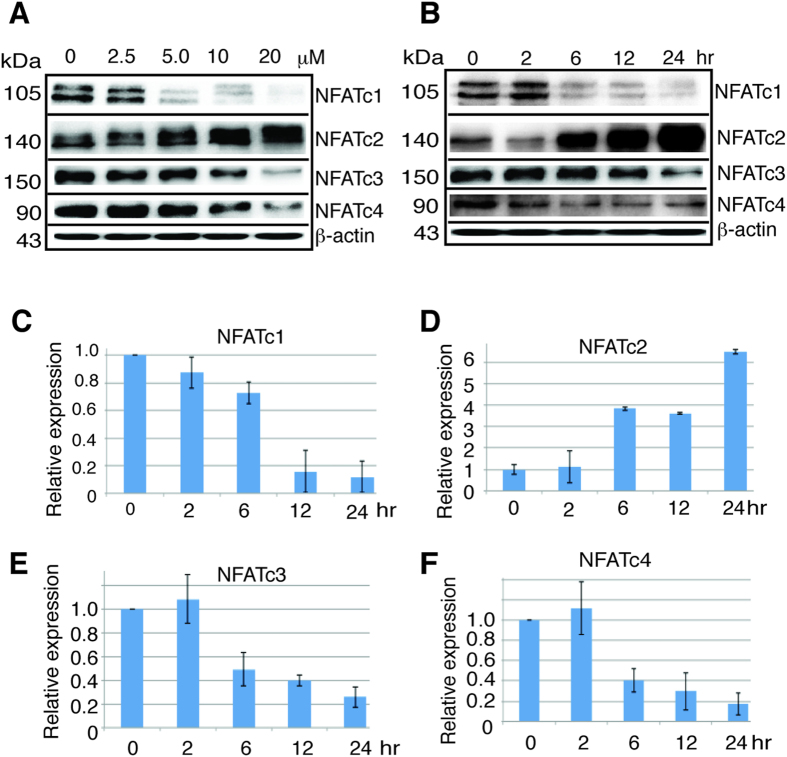
AS downregulates NFATc1, NFATc3, NFATc4 and c-Myc but upregulates NFATc2. (**A**) HCT116 cells were treated with AS in different concentration for 24 hours and blotted with antibodies as indicated. (**B**) HCT116 cells were treated with 10 μM AS from 2 to 24 hours and blotted with antibodies as indicated. (**C**–**F**) RT-PCR of NFATc1, NFATc2, NFATc3, and NFATc4 after HCT116 cells were treated with 10 μM AS for different time course. Error bar represents the SD of the results of three separate experiments. kDa, molecular weight.

We asked if expression of NFAT proteins would be regulated by AS by treating HCT116 cells with different concentration of AS from 2.5 μM to 20 μM for 24 hours. AS indeed inhibited the expression of NFATc1, NFATc3, and NFATc4 (Fig. 2A), the inhibition was apparent on NFATc1 with 5 μM and nearly complete with 20 μM, while the inhibition on NFATc3 and NFATc4 was apparent with 10 μM. Surprisingly AS stimulated NFATc2 expression (Fig. 2A), which was evident at 5 μM. Both the inhibition on NFATc1, NFATc3, and NFATc4, and the stimulation on NFATc2 were dose-dependent. Similar results were obtained in other solid tumor cell lines including AGS gastric cancer cells that also contain wild type p53 (Fig. S1) indicating these findings are not limited to HCT116. We examined the time course and treated HCT116 cells with 10 μM AS from 2 to 24 hours. The inhibition on NFATc1 and NFATc4 was apparent after 6 hours of AS treatment, and 12 hours for NFATc3, and for NFATc2 the stimulation was evident after 6 hours of AS treatment (Fig. 2B). These results indicate that AS exerted potent but differential effect on the expression of four classic NFAT proteins. To investigate the mechanism of this effect by AS on NFAT gene family, we took advantage of pan-caspase inhibitor Z-VAD-FMK and proteasome inhibitor MG132. We found z-VAD-FMK had no impact on the AS effect over NFATc1 and NFATc2 or c-Myc expression (Fig. S2A). MG132 showed no impact on the AS inhibition of NFATc1 expression (Fig. S2B), but blocked the AS stimulation of NFATc2 expression (Fig. S2C), suggesting that AS uses one mechanism to exert its inhibitory effect while another for its stimulatory effect. By performing RT-PCR to analyze the effect of AS on the mRNA transcription of NFAT genes, we found that AS markedly reduced the transcription of NFATc1, NFATc3, and NFATc4 (Fig. 2C,E,F), while dramatically stimulated the transcription of NFATc2 (Fig. 2D). These data demonstrated that the effect of AS on NFAT expression was at the transcriptional level.

### AS inhibited PML mRNA and protein expression while PML knockdown reduced NFATc1, NFATc2, NFATc3, NFATc4 and c-Myc

**Figure 3:**
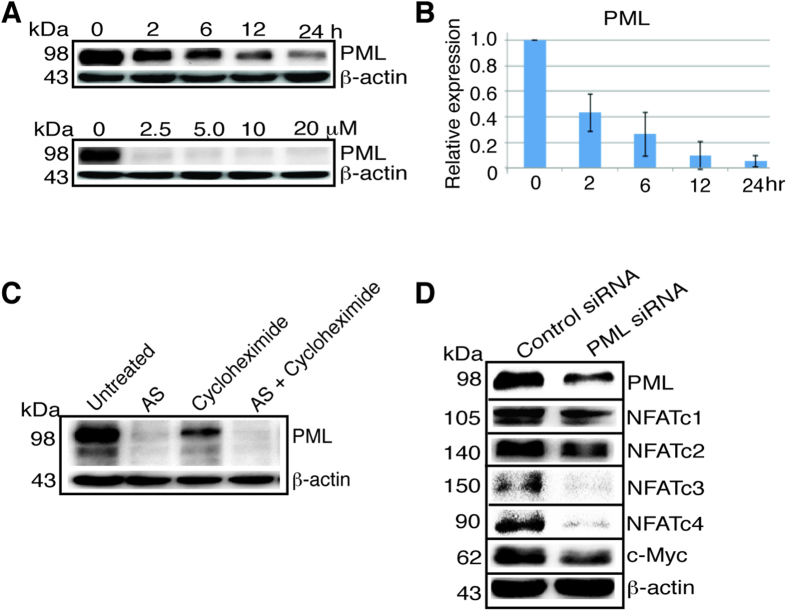
AS suppresses PML transcription while PML knockdown reduces NFATc1, NFATc2, NFATc3, NFATc4 and c-Myc expression. (**A**) PML western blot after treatment of HCT116 cells with 10 μM AS in different time course or different concentration as indicated for 24 hours. (**B**) RT-PCR of PML after treatment of HCT116 cells with 10 μM AS for different time course. Error bar represents the confidence interval of the results of three separate experiments. (**C**) HCT116 cells were left untreated or treated with 10 μM AS, 500 μM cycloheximide or both for 24 hours. (**D**) HCT116 cells were treated with a control siRNA or PML siRNA for 48 hours and blotted with the antibodies as indicated. kDa, molecular weight.

Since PML is directly targeted by ATO for degradation in APL, we asked if the arsenic effect on NFAT could be mediated by PML. PML was previously shown to activate NFATc1 expression in Jurkat and EL4 cells^24^. We found that AS potently inhibited the protein expression of PML in HCT116 cells (Fig. 3A upper panel). The inhibition of PML was observed as early as 2 hours after the treatment with 10 μM AS. With 2.5 μM or higher concentration for 24 hours AS nearly abolished PML protein expression (Fig. 3A lower panel). By RT-PCR AS showed similar inhibition on the mRNA expression of PML (Fig. 3B), indicating that AS regulates PML through transcription as well, in addition to its known effect through the proteasome^5^. This was also confirmed with decreased PML when HCT116 cells were treated with cycloheximide, a protein synthesis inhibitor, and with both cycloheximide and AS PML level was completely abolished (Fig. 3C). PML knockdown with siRNA lowered the expression of NFATc1, NFATc2, NFATc3, NFATc4, and c-Myc (Fig. 3D). This data indicates that AS differentially regulates NFAT expression, inhibiting all four classic NFAT proteins through repressing PML, but using a non-PML pathway to stimulate NFATc2 expression. In addition, such pathway must overcome the inhibitory effect of AS on PML to stimulate NFATc2.

### NFATc3 knockdown inhibited colon cancer cell growth *in vitro* and in xenograft

**Figure 4:**
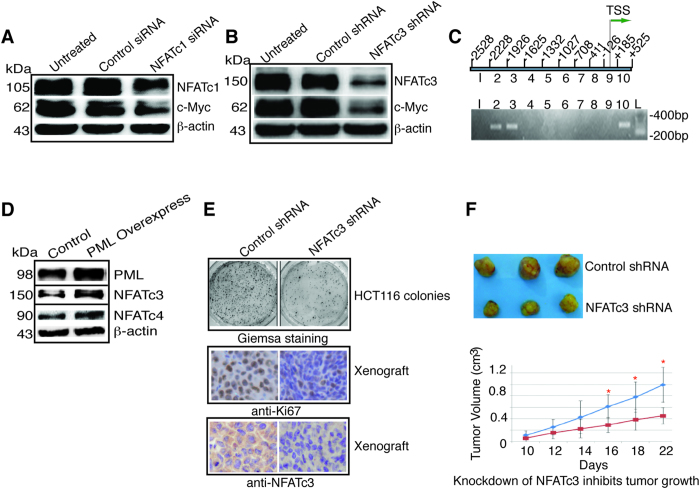
NFATc3 knockdown retards cell proliferation and tumor growth. (**A**) HCT116 cells were treated with control siRNA or NFATc1 siRNA for 48 hours, and blotted with the antibodies as indicated. (**B**) HCT116 cells were transfected with lentivirus carrying a control or NFATc3 shRNA for 96 hours, and blotted with the antibodies as indicated. (**C**) ChIP of c-Myc promoter using anti-NFATc3 antibody. Ten segments of c-Myc promoter were generated by PCR (upper panel) and subjected to ChIP (lower panel). NFATc3 antibody precipitated segment 2, 3, and 10. TSS, transcription start site. (**D**) HCT116 cells were transfected with the plasmid pCMV6-AC-GFP-PML for 24 hours and cell lysates were blotted with the antibodies indicated. (**E**) Giemsa staining of HCT116 colonies, anti-Ki67 and anti-NFATc3 immunostaining of xenograft at day-22 from nude mice containing lentivirus carrying a control or NFATc3 shRNA. (**F**) Knockdown of NFATc3 inhibits tumor growth. HCT116 cells (5 × 10^6^) transfected with lentivirus carrying a control or NFATc3 shRNA were injected into nude mice and tumor growth was measured every other day beginning day 10, statistical analysis of tumor volume between the control and NFATc3 shRNA group was performed (n = 10; blue = control; red = NFATc3 knockdown). Asterisk indicates statistically significant difference between the two groups. kDa, molecular weight.

NFATc1 was previously known to activate c-Myc^17^,^18^. Consistent with this, we found that in HCT116 cells knockdown of NFATc1 resulted in decreased expression of c-Myc (Fig. 4A). Knockdown of NFATc3 also markedly decreased c-Myc expression (Fig. 4B), indicating c-Myc is a target of NFATc3 as well. By chromatin IP (ChIP), we confirmed that NFATc3 indeed bond to the c-Myc promoter (Fig. 4C). While NFATc1 has been consistently shown to promote tumor growth, NFATc3 can be both stimulatory and inhibitory^16^,^23^. Since NFATc3 activates c-Myc, suggesting it serves as a stimulatory transcription factor for HCT116 cell growth. Indeed, overexpression of PML stimulated NFATc3 and NFATc4 expression (Fig. 4D), and knockdown of NFATc3 inhibited HCT116 colony formation (Fig. 4E, Giemsa staining) and proliferation as evidenced by the decreased Ki67 staining on the xenograft in nude mice (Fig. 4E, Ki67). In nude mice xenograft, tumors that carried NFATc3 shRNA showed persistent knockdown of NFATc3 as demonstrated by immunostaining at day 22 (Fig. 4E, anti-NFATc3) and significantly slower growth (Fig. 4F). These data indicate that NFATc3 promotes colon cancer cell growth and c-Myc is one of its target genes.

### AS stimulated NFATc2 expression by activating p53

**Figure 5:**
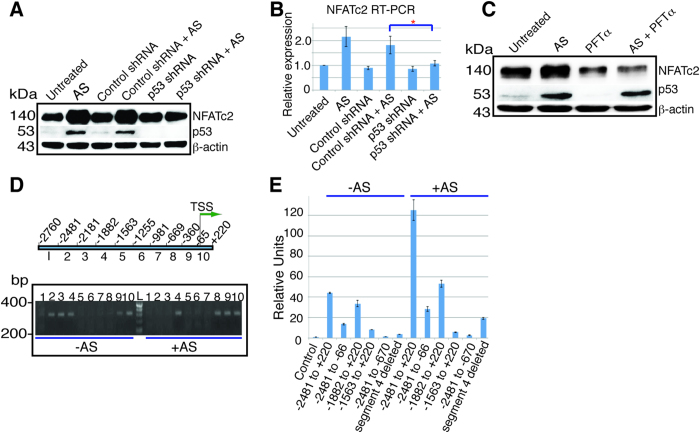
AS stimulates NFATc2 expression by activating p53. (**A**) HCT116 cells left untreated (Untreated), treated with AS 10 μM (AS), or infected with lentivirus carrying control shRNA for 96 hours (Control shRNA), then left untreated or treated with AS for 24 hours (Control shRNA + AS), or infected with lentivirus carrying p53 shRNA for 96 hours, then left untreated (p53 shRNA) or treated with AS for 24 hours (p53 shRNA + AS), and blotted with the antibodies as indicated. (**B**) RT-PCR of NFATc2 of HCT116 cells with the identical treatment. Data represents the result of one of the three similar experiments. *p < 0.05 indicates statistically significant difference between (Control shRNA + AS) and (p53 shRNA + AS). (*A*). (**C**) Western blot of NFATc2 and p53 in HCT116 cells left untreated or treated with AS, or PFTα 60 μM, or AS plus PFTα for 24 hours. PFTα was added one hour before AS. (**D**) NFATc2 promoter was separated into ten different segments by PCR as indicated and subjected to ChIP with anti-p53 antibody. In untreated HCT116 cells (−AS), p53 bond to the DNA sites on −2481 to −1564 (segments 2, 3, and 4) and −360 to +220 (segments 9 and 10). Upon AS stimulation (+AS), p53 bond to the sites on −1882 to −1564 (segment 4), and −669 to +220 (segments 8, 9 and 10). TSS, transcription start site. Numbers indicate relative position from the TSS. L = ladder. (**E**) Luciferase assay to determine p53 responsive sites on the NFATc2 promoter. HCT116 cells were transfected in triplicate with pCDNA3.1-p53 plasmid and an empty pGL3B vector as control (C), or pGL3B containing promoter sequence from −2481 to +220, from −2481 to −66, from −1882 to +220, from −1563 to +220, from −2481 to −670, and from −2481 to +220 with segment 4 deleted. Data represented the results without (−AS) or with (+AS) the treatment of AS for 12 hours. kDa, molecular weight. Error bar represents the confidence interval of the triplicate results. Nearly identical results were obtained with a repeated experiment.

The fact that AS stimulation of NFATc2 expression was at the mRNA level and was sensitive to a proteasome inhibitor suggested that a factor regulated by both proteasome and transcription may mediate this AS stimulatory effect, such as p53. Indeed, p53 was readily activated by AS in HCT116 cells (Fig. 5A) and AGS gastric cancer cells (Fig. S1), both of which contain wild type p53. We examined the cells that contain a mutant p53 including SW480 colon cancer cells, MGC803 gastric cancer cells and Panc-1 pancreatic cancer cells for NFATc2 expression after AS treatment. In these three cell lines, p53 expression was not stimulated by AS (Fig. S3), and NFATc2 was not changed by AS in SW480 cells but inhibited by AS in MGC803 and Panc-1 cells similar to that of NFATc1, NFATc3, and NFATc4 (Fig. S3). These results suggested that AS likely regulated NFATc2 through p53. Indeed, knockdown of p53 by an shRNA construct resulted in the loss of NFATc2 stimulation by AS (Fig. 5A,B). In addition, p53 inhibitor Pifithrin (PFTα) blocked the stimulation of NFATc2 expression by AS (Fig. 5C)^25^. These results suggest that NFATc2 is a direct target of p53.

### Regulation of NFATc2 promoter in basal and activated state by p53

The promoter recognition by p53 is dependent on the p53 responsive elements (PREs) that share sequence homology consisted of two decamers 5′-PuPuPuC(A/T)(T/A)GPyPyPy-3′ as well as DNA conformation^26^,^27^,^28^. For some genes, p53 recognizes a pentanucleotide 5′-(TGPyCC)_n_-3′ instead for transcription stimulation^26^,^27^. We analyzed the promoter region of NFATc2 for segments that might contain potential p53 binding sites using ChIP. We separated this promoter region into ten segments (Fig. 5D). NFATc2 promoter contains several PREs and pentanucleotides (Fig. S4). In the absence of AS, p53 bond to the segments 2, 3, 4, 9, and 10 (correspond to −2481 to −2182, −2181 to −1883, −1882 to −1564, −360 to −66, and −65 to +220). Upon stimulation with 10 μM AS for 12 hours, p53 binding sites were switched to the segments 4, 9, and 10 plus segment 8 (−669 to −361) (Fig. 5D). This indicates that p53 uses different combination of binding sites on the NFATc2 promoter to maintain the basal and the activated expression. To confirm that these binding sites indeed are critical to the stimulation of NFATc2 by p53 and AS, we performed luciferase assay using a p53-overexpressing plasmid. With the promoter comprising of −2481 to +220, there was marked stimulation of NFATc2-luciferase activity by p53, and this stimulation was further augmented by the addition of AS (Fig. 5E, column −2481 to +220 without AS {−AS} and with AS {+AS}). When both segments 2 and 3 were deleted, modest loss of p53 and AS stimulation was observed (Fig. 5E, column −1882 to +220 without AS and with AS), consistent with the ChIP data showing that these two segments served to maintain the basal level (Fig. 5D). The deletion of segments 2, 3 and 4 abolished much of the stimulation (Fig. 5E column −1563 to +220 without and with AS) and the deletion of segment 4 alone potently reduced p53 and AS stimulation (Fig. 5E, column segment 4 deleted without and with AS), indicating that the p53 binding sites on segment 4 were critical for p53 stimulation. This segment indeed contains several putative PREs and one pentanucleotide (Fig. S4). When segments 8, 9 and 10 were deleted, p53 and AS stimulations were nearly completely abolished (Fig. 5E, column −2481 to −670 without and with AS), in contrast to the more modest decrease when only segment 10 was deleted (Fig. 5E, column −2481 to −66 without and with AS), consistent with the ChIP showing the emergence of segment 8 upon AS stimulation, suggesting that the combination of the p53 binding sites on this segment possibly serves as a critical switch for p53 activation of NFATc2 expression. Consistent with this data, segment 8 contains two pentanucleotides and at least one PRE (Fig. S4). These data also indicate that p53 activates NFATc2 independent of PML as a transcriptional cofactor^11^.

### Reciprocal regulation of p53 and NFATc3, NFATc4, and NFATc2

**Figure 6:**
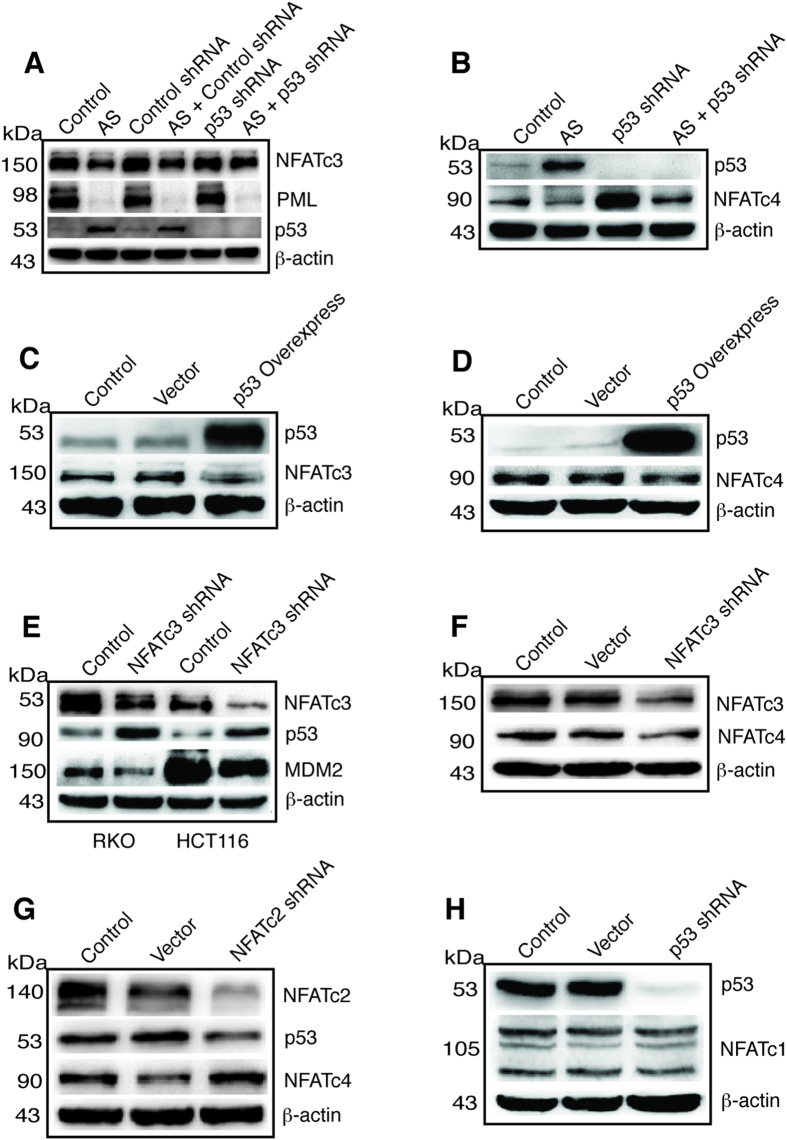
Reciprocal regulation of p53 and NFAT. (**A**) HCT116 cells were left untreated (Control) or treated with AS, or transfected with a control shRNA construct, or AS plus a control shRNA construct, p53 shRNA, or AS plus p53 shRNA. After 24 hours of treatment cell lysates were prepared and probed with antibody against NFATc3, PML, p53 and β-actin. (**B**) HCT116 cells were left untreated (Control) or treated with AS, or transfected with p53 shRNA, or AS plus p53 shRNA. After 24 hours of treatment cell lysates were prepared and probed with antibody against p53, NFATc4 and β-actin. (**C**) HCT116 cells were left untreated, or transfected with a empty vector, or with pcDNA3.1-p53 overexpression construct. After 24 hours of treatment cell lysates were prepared and probed with antibody against p53, NFATc3 and β-actin. (**D**) Same treatment as in C and lysates probed with antibody against p53, NFATc4 and β-actin. (**E**) RKO and HCT116 cells were untreated or transfected with a NFATc3 shRNA construct and lysates probed with antibody against p53, MDM2, NFATc3 and β-actin. (**F**) HCT116 cells were untreated or transfected with an empty vector or NFATc3 shRNA construct and lysates probed with antibody against NFATc3, NFATc4, and β-actin. (**G**) HCT116 cells were left untreated, transfected with an empty vector or NFATc2 shRNA construct, lysates were probed with antibody against NFATc2, p53, NFATc4, and β-actin. (**H**) HCT116 cells were left untreated, transfected with an empty vector or p53 shRNA construct, lysates were probed with antibody against p53, NFATc1 and β-actin. kDa, molecular weight.

To understand if p53 regulates PML, NFATc1, NFATc3, and NFATc4, we performed p53 knockdown experiment using a p53 shRNA construct. As shown in Fig. 6A, PML was suppressed by AS while p53 knockdown did not alter the basal level of PML or the effect of AS on PML. P53 knockdown did not change the basal level of NFATc3, however, it partially blocked the inhibitory effect of AS on NFATc3 (see AS+p53 shRNA lane), suggesting that p53 was partially required by AS to suppress NFATc3 expression. Moreover, p53 knockdown dramatically increased the basal level of NFATc4 and partially blocked the AS inhibition of NFATc4 and restored NFATc4 expression to the untreated basal level (Fig. 6B), indicating that p53 suppresses the basal expression of NFATc4 and that AS requires p53 for maximal suppression of NFATc4. Since AS inhibits PML but activates p53, how these two proteins work together to regulate NFAT proteins upon AS treatment remains unclear. PML is a positive regulator of all four classic NFAT proteins since its knockdown decreased their protein levels (Fig. 3D), while p53 suppresses NFATc3 and NFATc4 but activates NFATc2. This was confirmed by p53 overexpression that inhibited both NFATc3 (Fig. 6C) and NFATc4 (Fig. 6D). We next examined if NFATc3 reciprocally regulates p53. Indeed, NFATc3 knockdown increased the expression of p53 while decreasing the expression of MDM2 in both RKO and HCT116 cells (Fig. 6E). These results indicate that p53 is a target gene of NFATc3, consistent with the cell growth-promoting effect of NFATc3 *in vitro* and in xenograft (Fig. 4E,F) by suppressing p53. Consistent with NFATc4 being the target of p53, knockdown of NFATc3 showed decreased NFATc4 expression (Fig. 6F). We also knocked down NFATc2 with a shRNA construct and found that p53 expression was decreased, indicating that p53 was positively regulated by NFATc2 (Fig. 6G). Consistent with this, knockdown of NFATc2 resulted in increased expression of NFATc4 (Fig. 6G), as decreased p53 expression caused by NFATc2 knockdown would release NFATc4 from p53 suppression. In contrast, NFATc1 expression was not affected by p53 knockdown (Fig. 6H).

### Tumor expression of nuclear NFATc1 was associated with inferior survival while NFATc2 with superior survival in patients with colon cancer

**Figure 7:**
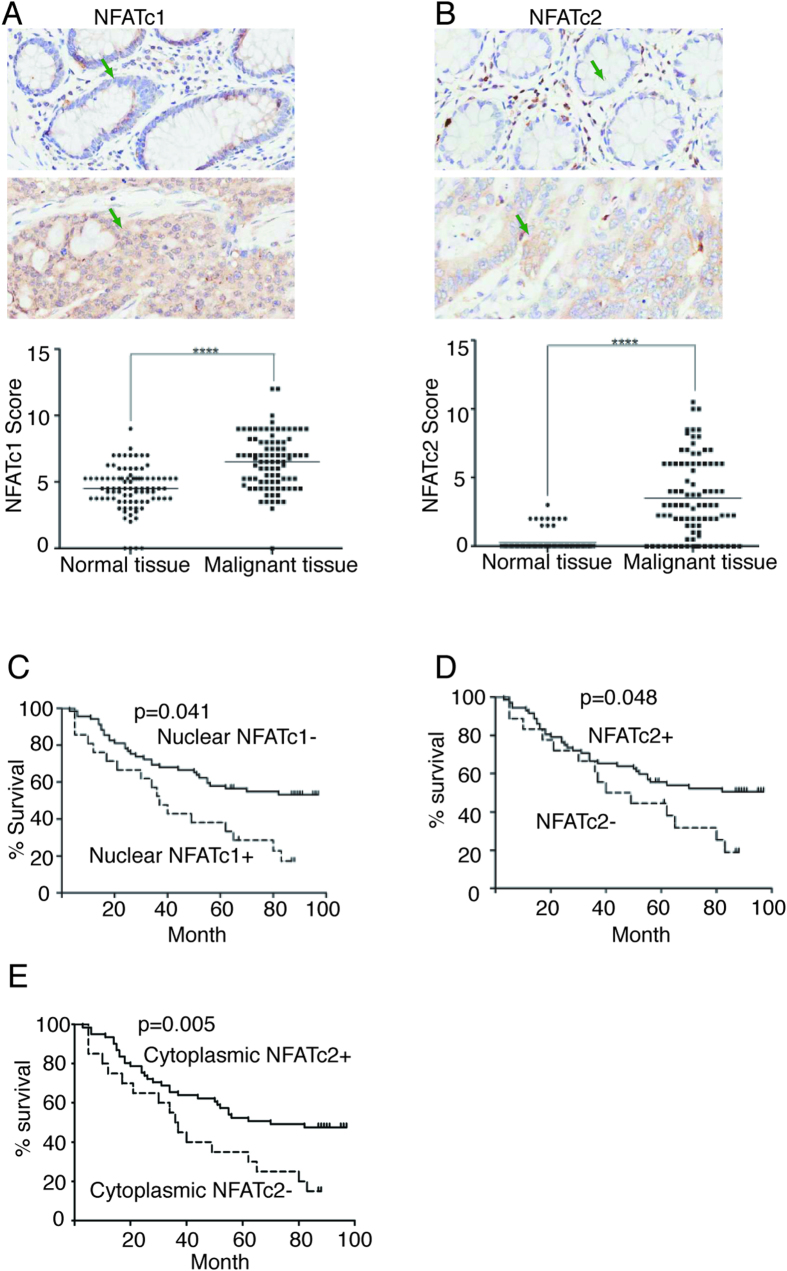
Tumor expression of NFATc2 and nuclear NFATc1 correlated with survival. (**A**) Expression of NFATc1 in adjacent normal colon endothelium (upper panel) and in colon cancer (middle panel), and comparative analysis of NFATc1 expression between the adjacent normal endothelium and colon cancer of the 90 cases (****p < 0.0001, lower panel). Green arrows point to representative cells with negative or positive expression. (**B**) Expression of NFATc2 in the adjacent normal colon endothelium (upper panel) and in colon cancer (middle panel), and comparative analysis of NFATc2 expression between the adjacent normal endothelium and colon cancer of the 90 cases. (****p < 0.0001, lower panel). Green arrows point to representative cells with negative or positive expression. (**C**) Kaplan-Meier analysis of overall survival by the function of NFATc1 nuclear expression. Statistically significant difference (p = 0.041) between the patients with negative and positive nuclear NFATc1 expression (NFATc1− or NFATc1+) is indicated. (**D**) Kaplan-Meier analysis of overall survival by the function of NFATc2 expression (positive expression in either cytoplasm or nucleus or both) in the 90 patients with colon cancer. Statistically significant difference (p = 0.048) between the patients with positive or negative NFATc2 expression (NFATc2+ or NFATc2-) is indicated. (**E**) Kaplan-Meier analysis of overall survival by the function of NFATc2 cytoplasmic expression. Statistically significant difference (p = 0.005) between the patients with positive and negative NFATc2 cytoplasmic expression (cytoplasmic NFATc2+ or cytoplasmic NFATc2−) is indicated.

The AS suppression of NFATc1 is consistent with NFATc1’s potential oncogenic role in colon cancer, while the stimulation of NFATc2 by p53 suggests NFATc2 may function as a tumor suppressor^22^. To determine if these laboratory observations provide clinical insight, we investigated the relationship of NFATc1 and NFATc2 tumor expression and the survival of patients with history of colon cancer. We selected a human colon cancer tissue array that contained a total of 90 cases with survival data and with adjacent normal colon tissue as control. This cohort of 90 cases recorded a median follow-up of approximately 84 months (see Materials and Methods and Table S2). We found that expression of NFATc1 and NFATc2 were both increased in the malignant tissues in comparison to the adjacent normal colon endothelium (Fig. 7A,B). NFATc1 expression was more extensive compared to NFATc2 in both nucleus and cytoplasm (Fig. 7A,B). All cases except one had positive cytoplasmic expression of NFATc1, and positive nuclear expression of NFATc1 was detected in 46 cases and not detected in 44 cases (Table S3). Kaplan-Meier analysis shows positive nuclear expression of NFATc1 was significantly associated with worse survival (Fig. 7C). The median survival in the 44 patients whose tumor was negative for nuclear NFATc1 expression was 69.8 months compared to 53.2 months in the 46 patients whose tumor showed positive nuclear NFATc1 expression (Table S3). NFATc2 expression was detected in 72 cases, and out of these 72 cases, 69 had cytoplasmic expression (Table S4). Kaplan-Meier analysis showed that the patients whose tumor was positive for NFATc2 expression had significantly better survival than the patients whose tumor was negative for NFATc2 (Fig. 7D). The median survival of these 72 cases was 64.5 months, significantly better than 49.4 months in the 18 patients whose tumor lacked NFATc2 expression (Table S4). When Kaplan-Meier analysis was performed based on cytoplasmic expression of NFATc2, the 69 patients whose tumor was positive for NFATc2 in the cytoplasm had significantly better survival than the 21 patients whose tumor was negative for NFATc2 in the cytoplasm (Fig. 7E), with the median survival being 66.6 months versus 44.8 months (Table S4). Because age and stage showed statistically significant impact on the survival of these colon cancer patients on univariate analysis, we performed multivariate analysis including these two factors to confirm if the impact of NFATc1 nuclear expression and NFATc2 cytoplasmic expression on the survival were still significant (Table S5). Positive NFATc2 cytoplasmic expression remained significantly associated with superior survival and positive nuclear NFATc1 expression remained significantly associated with inferior survival (Table S5). These data indicate that NFATc1 and NFATc2 played opposing role in the clinical outcome of the colon cancer patients. We also performed NFATc3 and NFATc4 immunostaining but found no correlation of their expression with patient survival (data not shown).

## Discussion

By studying the cytotoxic effect of AS in solid tumor cells, we have uncovered a novel mechanism of NFAT regulation by PML and p53. Further, we have shown that p53 and NFAT proteins form a complex reciprocal regulatory relationship. This discovery indicates that PML and p53 are integral elements for the regulation of cell growth and differentiation governed by NFAT pathway.

It was previously demonstrated that ATO inhibited PML by direct binding to PML protein and inducing its proteasome degradation^5^. Here, we have found that AS also regulates PML at the transcriptional level by potently suppressing its mRNA expression. This finding is significant as it indicates a potentially new mechanism for arsenic compounds to regulate cell growth and differentiation. Whether or not this transcriptional suppression of PML contributes to the arsenic killing of APL cells will be important to further investigate. Furthermore, knockdown of PML decreased the expression of all four classic NFAT proteins indicating PML is a positive regulator of all NFAT proteins and likely a key regulator of many biological processes modulated by NFAT. How PML regulates the pathologic and physiologic function of NFAT signaling will require further investigation. Because NFAT genes are involved in numerous pathologic and physiologic processes such as immune regulation, angiogenesis, cardiovascular development, bone homeostasis, neurologic development and many others^13^,^14^,^15^,^16^, PML likely plays a critical role in modulating the magnitude of the effect played by NFAT in these processes by acting as a transcription co-regulator to fine-tune the level of NFAT expression in response to physiological and pathological stimuli.

We also showed for the first time that AS activated p53 to stimulate the expression of NFATc2 by binding to the NFATc2 promoter: p53 appears to occupy a set of the binding sites (PREs) to maintain the basal NFATc2 expression, but upon AS treatment p53 switches to utilize a different combination of the binding sites to drive NFATc2 expression. This elegant modulation of NFATc2 expression by p53 is surprising as NFATc2 has been shown in several cellular context to stimulate cell growth^14^,^16^,^18^,^20^,^28^. However, previously NFATc2 was also shown to cause cell growth arrest in NIH3T3 cells and inhibit stat5 activity in breast cancer cells^22^,^29^. The direct stimulation of NFATc2 by p53 indicates a protective role of NFATc2 in preventing cancer progression. Consistent with this, our patient outcome data showed that tumor expression of NFATc2 was associated with significantly better survival. NFATc2 likely opposes the action of NFATc1, NFATc3, and NFATc4 in colon cancer cells as those tumors lacking NFATc2 expression most likely had a more aggressive clinical course driven in part by the unopposed actions of NFATc1, NFATc3, and NFATc4. NFATc1 has been shown to stimulate cell growth and invasion in several studies^19^,^20^,^21^,^22^. Recently, NFATc1 was found to be associated with metastatic capacity and worse survival of stage II and III colon cancer^30^. Consistent with this, our data showed that NFATc1 expression was detected in nearly all colon cancers in our cohort, and in approximately 50% of these cases NFATc1 was expressed in both nucleus and cytoplasm, and nuclear expression of NFATc1 was associated with worse survival. It would be intriguing to further understand the mechanism underlying the differential actions of NFAT proteins.

In addition to its positive regulation of NFATc2, p53 negatively regulates NFATc3 and NFATc4. NFATc3 reciprocally inhibits p53. Knockdown of p53 increased while overexpression of p53 suppressed the expression of NFATc3 and NFATc4. NFATc3 knockdown increased p53 expression and led to decreased NFATc4. Moreover, NFATc3 stimulates MDM2 expression as its knockdown led to decreased expression of MDM2. NFATc3 knockdown reduced cell proliferation *in vitro* and tumor growth in xenograft consistent with its cell growth-promoting effect. PML upregulates NFATc1, NFATc3, and NFATc4 as its knockdown reduced the level of expression of all NFAT proteins and c-Myc, suggesting that PML may promote cell growth in colon cancer cells, a notion different than the previous findings in the leukemia and myeloma cells in which PML functions as a tumor suppressor gene and cooperates with p53^7^,^8^,^9^,^10^. In our findings p53 suppression of NFATc3 and NFATc4 appears to oppose the action of PML. NFATc1 does not appear to be affected by p53 knockdown. Whether or not NFATc2 and NFATc3 directly regulate p53 promoter and if p53 suppresses NFATc3 and NFATc4 by direct promoter binding remain to be investigated. We recently discovered that AS and JQ1, a specific BET bromodomain inhibitor, exerted synergistic cell killing and p53 activation in solid tumor cells, unveiling a potential key mechanism that mediates the arsenic effect^31^.

**Figure 8:**
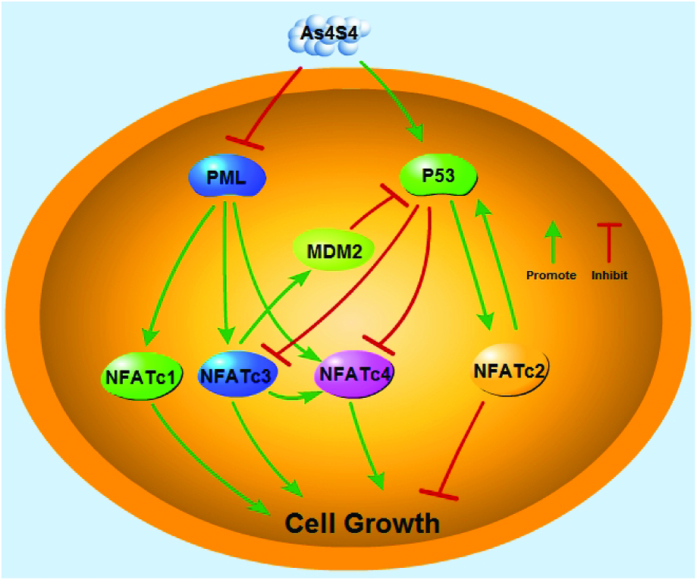
AS modulates NFAT signaling through PML and p53. AS suppresses NFATc1, NFATc3, and NFATc4 expression by repressing PML while inducing NFATc2 expression by activating p53. Activated p53 suppresses NFATc3 and NFATc4 while reciprocally NFATc3 stimulates MDM2 to suppress p53, leading to increased NFATc4 expression, thereby forming a negative feedback loop. NFATc2 positively regulates p53 expression while AS stimulates p53 to induce NFATc2, forming a positive feedback loop. The end outcome exerted by AS upon these complex regulations is the suppression of cell growth. Green arrow indicates stimulation; red stop sign indicates inhibition.

In summary, our study has revealed a complex yet elegant relationship among NFAT, PML and p53 pathways as illustrated in Fig. 8. These relationships include the stimulatory effect of PML on the expression of all four NFAT proteins that is differentially regulated by AS. AS suppresses PML to inhibit the expression of NFATc1, NFATc3 and NFATc4, while stimulates p53 to suppress NFATc3 and NFATc4 and activates NFATc2. NFATc2 positively regulate p53 while NFATc3 represses p53 expression, forming a positive feedback loop between NFATc2 and p53 while a negative feedback loop between NFATc3 and p53. Because AS potently stimulates p53 which likely overcomes its suppression on PML, leading to a net positive regulation of NFATc2 by p53. This is consistent with the previous finding that PML is not essential for p53 expression^12^. The complex relationships among NFAT, PML and p53 remain to be further explored. Because NFAT proteins regulate numerous physiologic and pathologic processes, the discovery of the role of PML and p53 in regulating NFAT expression provides a new direction for understanding the complex yet intriguing world of NFAT gene family.

## Materials and Methods

### Cell lines, animals, and reagents

HCT116, AGS, RKO, SW480, MGC803, and Panc-1 cell lines were obtained from the Chinese Academy of Sciences Committee Type Culture Collection cell bank (China). AGS and MGC803 cells were cultured in DMEM/F-12 1:1 medium (Thermo Fisher Scientific). HCT116, SW480, Panc-1 and RKO cells were grown in DMEM media (Hyclone, USA) at 37 °C and 5% CO_2_, supplemented with 10% fetal bovine serum (Gibco, USA). Nude mice BALB/c-nu (4 weeks of age, male, 16–18 g, n = 10) were purchased from Shanghai Laboratory Animal Research Center, maintained on standard chow and water. All experimental protocols were approved by the Ethics Committee of Xinhua Hospital Affiliated to Shanghai Jiao Tong University School of Medicine. All the methods were carried out according to the Ethics Committee of Xinhua Hospital Affiliated to Shanghai Jiao Tong University School of Medicine.

Reagents and antibodies. AS (As_4_S_4_) was generously provided by Shanghai Institute of Hematology, Rui Jin Hospital Affiliated to Shanghai Jiao Tong University School of Medicine. As_4_S_4_ was dissolved in PBS for 12 hours, filtered through a 0.22 micron filter, and its concentration was measured by Inductively Coupled Plasma Mass Spectrometer (ICP-MS) (7500a, Agilent, USA). 3-(4,5-dimethylthiazol-2-yl)-2,5-diphenyltetrazolium bromide (MTT), MG132, and Pifithrin α❒(PFTα) were purchased from Sigma-Aldrich, Z-VAD-FMK from Promega, EDTA-Trypsin from Gibco. Antibodies for c-myc, NFATc2 and p53 were purchased from Cell Signaling Technology, for NFATc1 (for western blotting) and NFATc3 from Santa Cruz Biotechnology, for NFATc4 and PML from Abcam, and for β-actin from Proteintech. Chromatin IP-Validated Monoclonal anti-p53 was purchased from Sigma-Aldrich. Anti-Ki67, HRP-labeled Goat Anti-Rabbit IgG and HRP-labeled Goat Anti-Mouse IgG were purchased from Beyotime.

### MTT assay

Cells (5 × 10^3^ cells/well, 90 μl) were plated into 96-well plates. Ten μl MTT (5 mg/ml) was added to each well and incubated at 37 °C for 4 hours, then 150 μl SDS was added to each well and incubated at 37 °C overnight. The absorbance at 562 nm was measured using a microplate reader (Bio-TEK, USA). Cells incubated without any treatment were used as control. Culture medium without cells was used as blank control. Each sample was assayed in quadruples. It was denoted that the percentage cell viability = (average OD of experimental group - average OD of blank control group/average OD of control group - average OD of blank control group) × 100%.

### Quantitative Real-Time RT-PCR

Total RNA was isolated from HCT116 cells using TRIzol reagent (Takara), and quantity was determined using a NanoDrop ND-1100 (NanoDrop Technologies). cDNA was synthesized using 1 μg of total RNA with the one-step Prime Script RT reagent kit (TaKaRa, Dalian, China). Transcript levels of cDNA were measured utilizing a 7500 Real-Time PCR System (Applied Biosystems, Foster City, CA, USA) with SYBR Premix Ex Taq (TaKaRa). GAPDH was used as an internal control for normalization. Please see Supplementary Information for the oligonucleotide primers used.

### Western blotting

Cell lysates were extracted using enhanced RIPA Lysis Buffer (Beyotime) containing 1% dilution of the Phenylmethanesulfonyl fluoride (PMSF) (Beyotime). Protein concentrations were determined using a microplate reader (Bio-TEK, USA) with the enhanced BCA Protein Assay kit (Beyotime). Equal amounts of protein in each lane were separated by 8–10% SDS-PAGE and transferred to a 0.45 μm PVDF membrane (Millipore, Billerica, MA, USA). After blocking the membrane in 5% nonfat-milk, the membrane was incubated with primary antibodies at 4 °C overnight, followed by incubating with labeled secondary antibody at room temperature for 1 hour, washed, added Immobilon Western Chemilum HRP Substrate (Millipore, USA) and image was acquired using GelDoc XR System (Bio-rad, USA).

### Transfection, shRNA-containing lentivirus infection, and siRNA

Plasmid (pCMV6-AC-GFP) carrying PML was purchased from ORIGENE. Plasmid (pCDNA3.1) carrying P53 was purchased from Genechem (China). To overexpress PML or p53, HCT116 cells were cultured in a 6-well plate to 75–85% confluence. A mixture of 10 μl Lipofectamine 2000 reagent (Invitrogen) and 4 μg plasmid DNA was used to transfect each well in the absence of serum. After 6 hours, the medium was replaced with 10% FBS DMEM for additional 18 hours before analysis. Lentiviruses (hU6-MCS-CMV-EGFP) carrying an shRNA targeting p53 (shp53) or a control shRNA were gifts from H. Chen laboratory at Xinhua Hospital Affiliated to Shanghai Jiao Tong University School of Medicine. Lentiviruses (pHBLV-U6-Zsgreen) carrying NFATc2 (shNFATc2) and NFATc3 shRNA (shNFATc3) were provided by HanBio (China). To knock down p53, NFATc2, or NFATc3, HCT116 cells were grown to 30–40% confluency in 6-well culture plates and then infected with lentivirus carrying shRNA. The infection efficiency was monitored by fluorescence microscopy for GFP expression and also confirmed by protein level of the targeted gene. Approximately 90% infection efficiency was confirmed in each experiment (Fig. S5). After 10 hours of infection at 37 °C, cells were washed and incubated with fresh medium for additional 86 hours before analysis (medium was replaced daily). For siRNA, HCT116 cells were transiently transfected with a siRNA targeting PML (siPML) (Genechem, China) or NFATc1 (Santa Cruz Biotechnology) or a control siRNA (Genechem, China) in Opti-MEM (Invitrogen) using lipofectamine 2000. The transfection solution was removed from the cells and replaced with fresh medium after 6 hours, and cells were incubated for additional 42 hours before harvest. Please see Supplementary Information for the shRNA and siRNA sequences.

### Colony formation and xenograft experiment

HCT116 cells were trypsinized to generate a single-cell suspension, and 2000 cells/dish were seeded into 10 cm dishes. Dishes were returned to the incubator for two weeks, and the colonies were fixed with 4% paraformaldehyde for 15 min at room temperature and then stained with Giemsa staining fluid for 30 minutes. For xenograft, 5 × 10^6^ HCT116 cells expressing shNFATc3 (NFATc3 shRNA) were subcutaneously injected into the flank of nude mice, while the same number of HCT116 cells containing control shRNA was injected into the opposite flank of the same mice as a control. Growth curves were plotted based on mean tumor volume within each experimental group at the indicated time point. Tumor dimensions were measured every 2 days using a digital caliper, and the tumor volume was calculated using the following formula: V = ½(larger diameter) × (smaller diameter) × (smaller diameter)^32^. The expression of Ki67 and NFATc3 in the tissues was detected by immunohistochemical staining.

### Chromatin immunoprecipitation (ChIP) assay

ChIP assays were performed with the Agarose ChIP kit (Pierce, USA) according to the manufacturer’s instructions. After treatment with or without 10 μM As_4_S_4_ for 12 hours, HCT116 cells were subjected to cross-linking with 1% formaldehyde. Glycine solution was then added and DNA was sonicated to an average size of 100–300 base pair by an ultrasonic cell disruptor (Diagenode, Belgium). Co-immunoprecipitation was performed overnight at 4˚C with ChIP-Validated Monoclonal Anti-p53 (Sigma-Aldrich) or anti-NFATc3 by incubation at 4 °C for overnight on a rocking platform. Precipitates were washed and samples were extracted twice with elution buffer, heated at 65 °C to reverse crosslinks and DNA fragments were purified with phenol/chloroform and suspended in normal saline and used for RT-PCR. Please see Supplementary Information for the primer sequences.

### Luciferase reporter assays

HCT116 cells were seeded at the density of 2 × 10^5^/well in 48-well plates, NFATc2-luciferase reporter was cotransfected with pCDNA3.1-p53 plasmid using Lipofectamine 2000. Cells were incubated at 37 °C with 5% CO_2_ for 24 hours. After replacement with fresh medium, cells were treated without or with 10 μM As_4_S_4_ for 12 hours. Luciferase activities were determined using a Dual-Glo Luciferase Assay System (Promega) in the SpectraMax M5 (USA).

### Human colon cancer TMAs, immunohistochemical staining and scoring

The tissue microarrays (TMAs) containing 90 human colon cancer samples and adjacent normal colon tissues were purchased from Shanghai Outdo Biotech Co., Ltd. Median followup for this cohort of patients was approximately 8 years. The complete data of patient characteristics was available for all except two cases (Table S2). Immunohistochemical stains were performed according to the manufacturer’s protocol. Briefly, the tissue was deparaffinized, rehydrated, and pretreated for a heat-induced antigen retrieval step in sodium citrate (PH 5.96). Endogenous peroxidase activity was blocked by 0.3% H_2_O_2_ in methanol. After washing in phosphate-buffered saline, the samples were incubated 0.5 hours at 37 °C with a 1:6000 dilution for NFATc1 antibody (Sigma), and 1:500 dilution for NFATc2 antibody (Cell Signaling Technology, # 5861). After three washes with PBS, sections were incubated with secondary antibody (EnVision™+/HRP anti-rabbit/mouse immunohistochemistry kit, Dako). Sections were counterstained with hematoxylin. Two independent investigators unfamiliar with the clinico-pathological data determined the immunoreactivity score (IRS) for all the cases. For each case, three random fields were selected for scoring and a final average score of each slide was calculated in the final analysis. The staining results were evaluated based on the intensity of the staining and the percentage of cells stained positive^33^,^34^. For the intensity evaluation, it was based on four levels: level 0, no staining; level 1, weak staining detected; level 2, moderate staining; level 3, strong staining detected. For the percentage of positive cells stained, 0 indicates 0–4% percentage of positive cells, 1 indicates 5–24%, 2 indicates 25–49%, 3 indicates 50–74%, and 4 indicates 75–100%. Final IRS was determined as the product of the score of percentage of positive cells stained and the intensity score (ranging from 0 to 12), For NFATc1, an IRS ≤ 2 is considered negative and IRS > 2 considered positive. For NFATc2, IRS = 0 is considered negative, and IRS > 0 is considered positive.

### Statistical Analysis

Statistical difference between the relative intensity of NFATc1, NFATc2 staining in tumor sections and adjacent normal tissues was examined by the Wilcoxon signed rank test. The rest of continuous data were evaluated using independent T-test and paired T test. The association of NFATc1 and NFATc2 IRS in the malignant tissues with various clinico-pathological parameters was tested by Chi-square (X^2^). Overall survival was analyzed with Kaplan-Meier curves (from date of surgery to death); differences in survival among patient subgroups were analyzed using the Log-Rank test. Seven patients were lost to follow-up after 64–67 months (2 cases at 64, 4 cases at 65, and 1 case at 67 months). COX regression was used for univariate and multivariate survival analysis. Multivariate survival analysis was performed on all factors that were found to be significant in univariate analysis. p < 0.05 was considered statistically significant (all, 2-tailed). SPSS software (version 19.00) was used for statistical analysis.

## Additional Information

**How to cite this article**: Ding, W. *et al.* Study of Arsenic Sulfide in Solid Tumor Cells Reveals Regulation of Nuclear Factors of Activated T-cells by PML and p53. *Sci. Rep.* **6**, 19793; doi: 10.1038/srep19793 (2016).

## Supplementary Material

Supplementary Information

## References

[b1] Shen Z. X. *et al.* All-trans retinoic acid/As2O3 combination yields a high quality remission and survival in newly diagnosed acute promyelocytic leukemia. Proc Natl Acad Sci USA 101, 5328–35 (2004). 15044693 10.1073/pnas.0400053101 PMC397380

[b2] Chen S. J. *et al.* From an old remedy to a magic bullet: molecular mechanisms underlying the therapeutic effects of arsenic in fighting leukemia. Blood 117, 6425–6437 (2011). 21422471 10.1182/blood-2010-11-283598 PMC3123014

[b3] Wu T. *et al.* Tetra-Arsenic Tetra-Sulfide Containing Triple-Agent Regimen as the First Line Therapy for Acute Promyelocytic Leukemia: Expeditiously Consecutive Complete Remission and Improved Disease-Free Survival. The American Society of Hematology Annual Meeting, New York. Washington D.C.: B*lood* **110**, 591 (2007, November 16^th^).

[b4] Emadi A. & Gore S. D. Arsenic Trioxide – An Old Drug Rediscovered. Blood 24, 191–199, doi: 10.1016/j.blre.2010.04.001 (2010). PMC2918685 20471733

[b5] Zhang X. W. *et al.* Arsenic trioxide controls the fate of the PML-RARalpha oncoprotein by directly binding PML. Science 328, 240–243 (2010). 20378816 10.1126/science.1183424

[b6] Mao J. H. *et al.* As_4_S_4_ targets RING-type E3 ligase c-CBL to induce degradation of BCR-ABL in chronic myelogenous leukemia. Proc Natl Acad Sci USA 107, 21683–21688 (2010). 21118980 10.1073/pnas.1016311108 PMC3003020

[b7] Platanias L. C. Biological responses to arsenic compounds. J Biol Chem 284, 18583–18587 (2009). 19363033 10.1074/jbc.R900003200 PMC2707240

[b8] Guo A. *et al.* The function of PML in p53-dependent apoptosis. Nat Cell Biol 2, 730–736 (2000). 11025664 10.1038/35036365

[b9] Bernardi R. *et al.* PML regulates p53 stability by sequestering Mdm2 to the nucleolus. Nat Cell Bio 6, 665–672 (2004). 15195100 10.1038/ncb1147

[b10] Pearson M. *et al.* PML regulates p53 acetylation and premature senescence induced by oncogenic Ras. Nature 406, 207–210 (2000). 10910364 10.1038/35018127

[b11] de Stanchina E. *et al.* PML is a direct p53 target that modulates p53 effector functions. Mol Cell 13, 523–535 (2004). 14992722 10.1016/s1097-2765(04)00062-0

[b12] Joe Y. *et al.* ATR, PML, and CHK2 play a role in arsenic trioxide-induced apoptosis. J Biol Chem 281, 28764–28771 (2006). 16891316 10.1074/jbc.M604392200

[b13] Crabtree G. R. & Olson E. N. NFAT signaling: choreographing the social lives of cells. Cell 109, S67–79 (2002). 11983154 10.1016/s0092-8674(02)00699-2

[b14] Graef I. A., Chen F., Chen L., Kuo A. & Crabtree G. R. Signals transduced by Ca(2+)/calcineurin and NFATc3/c4 pattern the developing vasculature. Cell 105, 863–875 (2001). 11439183 10.1016/s0092-8674(01)00396-8

[b15] Winslow M. M. *et al.* Calcineurin/NFAT signaling in osteoblasts regulates bone mass. Dev Cell 10, 771–782 (2006). 16740479 10.1016/j.devcel.2006.04.006

[b16] Pan M. G., Xiong Y. & Chen F. NFAT gene family in inflammation and cancer. Curr Mol Med 13, 543–554 (2013). 22950383 10.2174/1566524011313040007 PMC3694398

[b17] Buchholz M. *et al.* Overexpression of c-myc in pancreatic cancer caused by ectopic activation of NFATc1 and the Ca2+/calcineurin signaling pathway. EMBO J 15, 3714–3724 (2006). 16874304 10.1038/sj.emboj.7601246 PMC1538549

[b18] Koenig A. *et al.* NFAT-induced histone acetylation relay switch promotes c-Myc-dependent growth in pancreatic cancer cells. Gastroenterology 138, 1189–1199.e1-2 (2010). 19900447 10.1053/j.gastro.2009.10.045 PMC2895621

[b19] Gregory M. A. *et al.* Wnt/Ca2+/NFAT signaling maintains survival of Ph+ leukemia cells upon inhibition of Bcr-Abl. Cancer Cell 18, 84–87 (2010). 10.1016/j.ccr.2010.04.025 PMC2904512 20609354

[b20] Foldynova-Trantirkova S. *et al.* Breast cancer-specific mutations in CK1epsilon inhibit Wnt/beta-catenin and activate the Wnt/Rac1/JNK and NFAT pathways to decrease cell adhesion and promote cell migration. Breast Cancer Res 12, R30 (2010). 20507565 10.1186/bcr2581 PMC2917022

[b21] Courtwright A. *et al.* Secreted frizzle-related protein 2 stimulates angiogenesis via a calcineurin/NFAT signaling pathway. Cancer Res 69, 4621–4628 (2009). 19458075 10.1158/0008-5472.CAN-08-3402 PMC2699405

[b22] Robbs B. K., Cruz A. L., Werneck M. B., Mognol G. P. & Viola J. P. Dual roles for NFAT transcription factor genes as oncogenes and tumor suppressors. Mol Cell Biol 28, 7168–7181 (2008). 18809576 10.1128/MCB.00256-08 PMC2593389

[b23] Glud S. Z. *et al.* A tumor-suppressor function for NFATc3 in T-cell lymphomagenesis by murine leukemia virus. Blood 106, 3546–3552 (2005). 16051745 10.1182/blood-2005-02-0493 PMC1895049

[b24] Lo Y. H., Wu C. C., Shih H. M. & Lai M. Z. Selective activation of NFAT by promyelocytic leukemia protein. Oncogene 27, 3821–3830 (2008). 18246125 10.1038/onc.2008.2

[b25] Komarov P. G. *et al.* A chemical inhibitor of p53 that protects mice from the side effects of cancer therapy. Science 285, 1733–1737 (1999). 10481009 10.1126/science.285.5434.1733

[b26] Menendez D., Unga A. & Resnick M. A. The expanding universe of p53 targets. Nat Rev Cancer 9, 724–737 (2009). 19776742 10.1038/nrc2730

[b27] Contente A., Dittmer A., Koch M. C., Roth J. & Dobbelstein M. A polymorphic microsatellite that mediates induction of PIG3 by p53. Nat. Genet. 30, 315–320 (2002). 11919562 10.1038/ng836

[b28] el-Deiry W. S., Kern S. E., Pietenpol J. A., Kinzler K. W. & Vogelstein B. Definition of a consensus binding site for p53. Nat Genet 1, 45–49 (1992). 1301998 10.1038/ng0492-45

[b29] Zheng J. *et al.* Negative cross talk between NFAT1 and Stat5 signaling in breast cancer. Mol Endocrinol 25, 2054–2064 (2011). 21964595 10.1210/me.2011-1141 PMC3231824

[b30] Tripathi M. K. *et al.* Nuclear factor of activated T-cell activity is associated with metastatic capacity in colon cancer. Cancer Res 74, 6947–6957 (2014). 25320007 10.1158/0008-5472.CAN-14-1592 PMC4252979

[b31] Zhang L., Tong Y. Y., Zhang X. L., Pan M. & Chen S. As_4_S_4_ combined with JQ1, cisplatin, or celecoxib inhibit gastric and colon cancer cell growth. Drug Des Devel Ther 9, 5851–5862, doi: 10.2147/DDDT.S92943 (2015). PMC4634829 26586936

[b32] Naito S., von Eschenbach A. C., Giavazzi R. & Fidler I. J. Growth and metastasis of tumor cells from a human renal cell carcinoma implanted into different organs of nude mice. Cancer Res 46, 4109–4115 (1986). 3731078

[b33] Ma Y., Ma L., Guo Q. & Zhang S. Expression of bone morphogenetic protein-2 and its receptors in epithelial ovarian cancer and their influence on the prognosis of ovarian cancer patients. J Exp Clin Cancer Res 29, 85 (2010). 20587070 10.1186/1756-9966-29-85 PMC2907340

[b34] Hirsch F. R. *et al.* Epidermal growth factor receptor in non–small-cell lung carcinomas: correlation between gene copy number and protein expression and impact on prognosis. J Clin Oncol 22, 3646–3654 (2003). 10.1200/JCO.2003.11.069 12953099

