# Supplementary material for: Study of Arsenic Sulfide in Solid Tumor Cells Reveals Regulation of Nuclear Factors of Activated T-cells by PML and p53
Source: Sci Rep. 2016 Jan 22;6:19793. doi: 10.1038/srep19793 (PMC4726130; doi:10.1038/srep19793)
Supplement: Supplementary Information [file srep19793-s1.pdf]

Study of Arsenic Sulfide in Solid Tumor Cells Reveals Regulation of Nuclear Factors of Activated T-cells by PML and p53

Wenping Ding[1*], Yingying Tong[1*], Xiuli Zhang[1], Minggui Pan[2,3], Siyu Chen[1]

[1]Department of Oncology, Xin Hua Hospital Affiliated to Shanghai Jiao Tong University School of Medicine, Shanghai, China

[2]Department of Oncology and Hematology, Kaiser Permanente Medical Center, Santa Clara, CA, and [3]Kaiser Permanente Division of Research, Oakland, CA, USA

Address correspondence to: Siyu Chen, Department of Oncology, Xin Hua Hospital Affiliated to Shanghai Jiao Tong University School of Medicine, Shanghai 200092, China, Phone +86-21-25077642

email: siyu.chen@shsmu.edu.cn

or Minggui Pan, Department of Oncology and Hematology, Kaiser Permanente Medical Center, 710 Lawrence Expressway, Santa Clara, CA 95051, USA

Phone: 1 408 851 4306

email: minggui.pan@kp.org

Supplemental Information

Materials and Methods

The oligonucleotide primers used for RT-PCR are as follows:

GAPDH: F (forward): 5’-ggcacagtcaaggctgagaatg-3’

R (reverse): 5’-atggtggtgaagacgccagta-3’

c-Myc:

F: 5’-cctccactcggaaggactatc-3’

R: 5’-tgttcgcctcttgacattctc-3’

NFATc1:

F: 5’-ggagatggaagcgaaaactg-3’

R: 5’-gcgggaaggtaggtgaaac-3’

NFATc2:

F: 5’-cacggggcagaactttacat-3’

R: 5’-ctgggactgggtggtaggta-3’

NFATc3:

F: 5’-cacaccactttgcttaccacat-3’

R: 5’-ccgttctgggtcatttatctgt-3’

NFATc4:

F: 5’-cttcccttcccagagtgatg-3’

R: 5’-accttcctccagcgtgatac-3’

PML:

F: 5’-agcacctccaaggcagtct-3’

R: 5’-gagttttcggcatctgagtctt-3’

The shRNA and siRNA sequences:

For p53, 5’-GACTCCAGTGGTAATCTAC-3’;

for NFATc2, 5’-GCCCATGGTTGAAAGACAAGA-3’;

for NFATc3, 5’-GCCAGATGATTGTGCATCCAT-3’;

for control, 5’-TTCTCCGAACGTGTCACGT-3’.

The sequence of siRNA for PML (siPML): 5’- ATACAACGACAGCCCAGAA-3’; and for negative control: 5’-TGAAGGAGTTCCTGATCTTTT-3’.

ChIP primers for NFATc2 and c-Myc promoter:

Ten sets of primers were designed to cover the NFATc2 promoter from -2760 to +220 from the transcription start site (TSS).

F1: 5′-GCCCCTGCCTTAGTGGATCTC-3′

R1: 5′-GGAGAAAAGCTAGGAATGCCT-3′

F2: 5′-AGGCATTCCTAGCTTTTCTCC-3′

R2: 5′- CTCAGGAATCATTTCAGAGTC-3′

F3: 5′- GACTCTGAAATGATTCCTGAG-3′

R3: 5′- TCCCTTCATCATGCTTATGCC-3′

F4: 5′-GGCATAAGCATGATGAAGGGA-3′

R4: 5′-TTAGTAGAGATGGGGTTTTG-3′

F5: 5′-GGGCGTGGTGGCTCACACCTG-3′

R5: 5′-CCCACTGAAGCAGAATTCCTA-3′

F6: 5′-TAGGAATTCTGCTTCAGTGGG-3′

R6: 5′-TAAGTCTGAATACAGACTCGT-3′

F7: 5′-ACGAGTCTGTATTCAGACTTA-3′

R7: 5′-TCTGCTTATGGGGCCAGGAGA-3′

F8: 5′-TCTCCTGGCCCCATAAGCAGA-3′

R8: 5′-CCATGGGGTGGAAGGGGGTAT-3′

F9: 5′-ATACCCCCTTCCACCCCATGG-3′

R9: 5′-CGTGGAGGCGGGGACAGGGCG-3′

F10: 5′- CGCCCTGTCCCCGCCTCCACG-3′

R10: 5′-CGGGAAGGCTGCGGGGCCGG-3′

Ten sets of primers were designed to cover the c-Myc promoter from -2528 to +525 from the TSS:

F1：CTTTGATTGTGCCACTGCACT

R1：TCACCACTGCTAATGAACATC

F2：GATGTTCATTAGCAGTGGTGA

R2：TGGGCAGCACCCGCGTTCAGG

F3：CCTGAACGCGGGTGCTGCCCA

R3：CCAAAGGCATTTTAAGTAAAC

F4：GTTTACTTAAAATGCCTTTGG

R4：GGAGTCTTGAGCTAATTAAAA

F5：TTTTAATTAGCTCAAGACTCC

R5：TTTGGGAGAAATCAAAGGTGC

F6：GCACCTTTGATTTCTCCCAAA

R6：TCTGGATTGGATACCTTCCAC

F7：GTGGAAGGTATCCAATCCAGA

R7：CCACGCGCGTACCAGGCTGCA

F8：TGCAGCCTGGTACGCGCGTGG

R8：GGCGCTTATGGGGAGGGTGG

F9：CCACCCTCCCCATAAGCGCC

R9：CGCTCGCTCCCTCTGCCTCT

F10：AGAGGCAGAGGGAGCGAGCG

R10：CGTCTAAGCAGCTGCAAGGAG

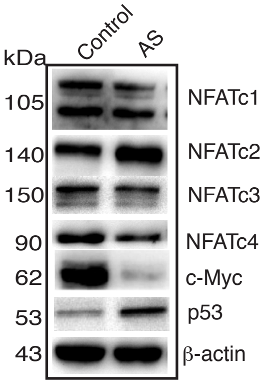

Figure S1: AS modulates NFAT, c-Myc and p53 expression in AGS cells.
Western blot of NFATc1-4, c-Myc and p53 after 1.0 μM AS treatment for 24 hours. β-actin as loading control.

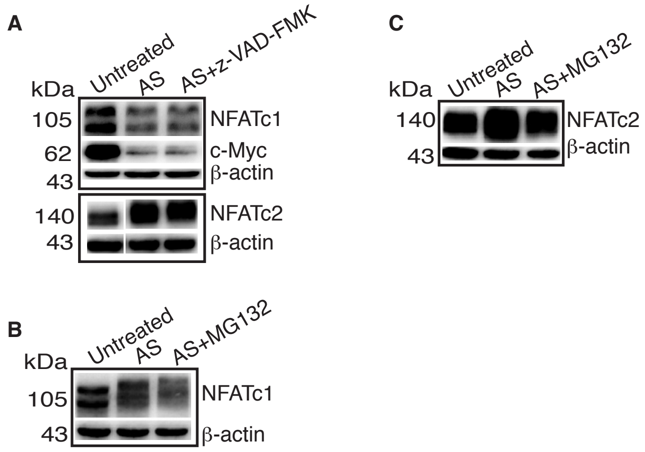

Figure S2: Impact of caspase inhibitor and proteasome inhibitor on NFATc1 and NFATc2.
A. z-VAD-FMK is a pan-caspase inhibitor and showed no impact on the effect of AS on NFATc1, NFATc2 or c-Myc. **B.** MG132 is a proteasome inhibitor, it did not impact the AS inhibition on NFATc1. **C.** MG132 blocked the AS stimulation of NFATc2. kDa, molecular weight.

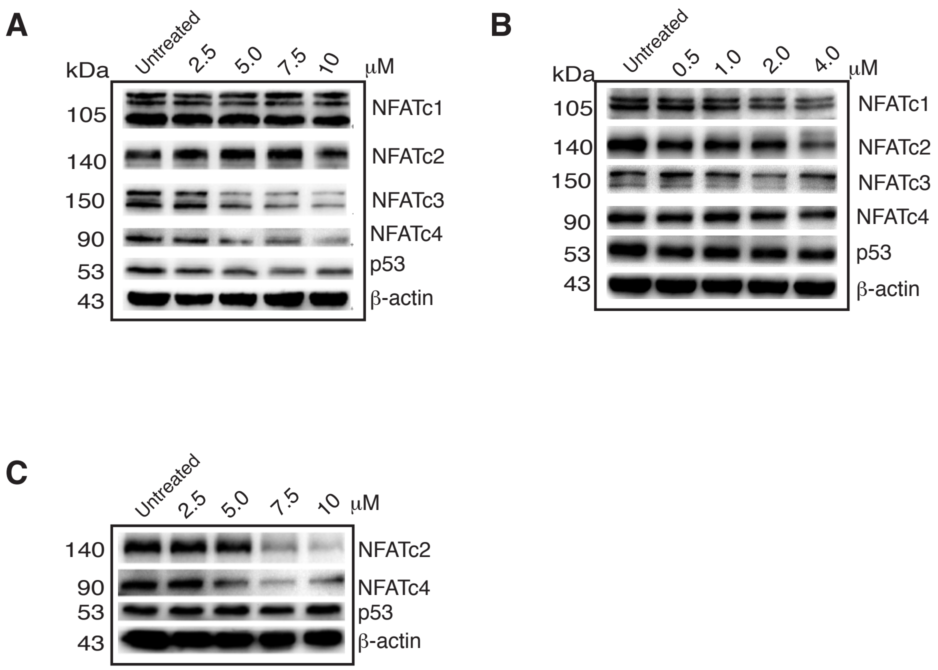

Figure S3: AS modulates NFATc1, c2, c3 and c4 (NFATc1-4) in other solid tumor cells.
A. Western blot of NFATc1-4 and p53 after AS treatment of SW480 colon cancer cells for 24 hours with indicated AS concentration. **B.** Western blot of NFATc1-4 and p53 after AS treatment of MGC803 gastric cancer cells for 24 hours. **C.** Western blot of NFATc2, NFATc4 and p53 after AS treatment of Panc-1 pancreatic cancer cells for 24 hours. AS concentration in μM. β-actin as loading control.

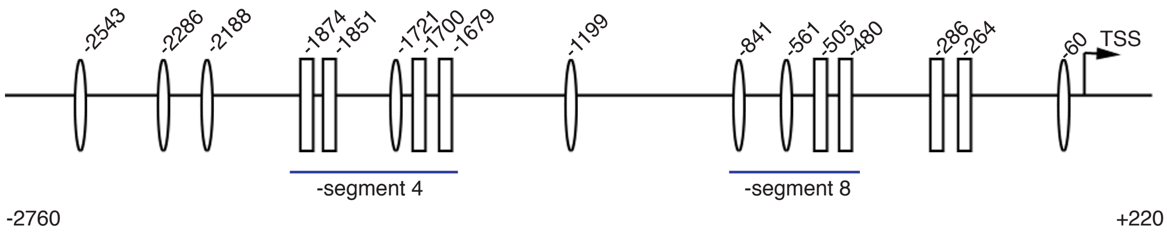

Figure S4: Putative p53 binding sites on the NFATc2 prooter region from -2760 to +220. TSS, transcription start site; Ovals indicatethe locations of pentanucleotides (TGCCC or TGTCC); Rectangles indicate the locations of the putative p53 responsive elements (PREs). The approximate positions of segment 4 and 8 are indicated (see Fig. 5D).

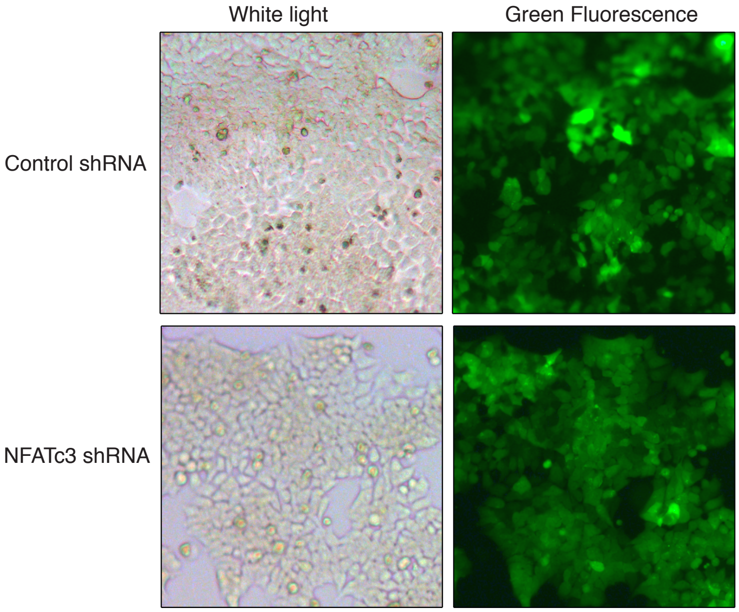

Figure S5. Monitoring shRNA-containing lentivirus infection efficiency by GFP expression

AS and CsA 24h viability	C1	A2.5	C1+A2.5	C5	A5	C5+A5	C1+A5	C5+A2.5
Plate 1	0.8240	0.8910	0.6910	0.7030	0.8060	0.3257	0.3844	0.5701
Plate 2	0.8073	0.8991	0.5894	0.6571	0.7661	0.3268	0.3773	0.5562
Plate 3	0.7917	0.9779	0.6605	0.7328	0.8689	0.3260	0.4081	0.5576
Plate 4	0.8558	0.8676	0.5981	0.6986	0.8203	0.3333	0.4102	0.5626
AS + CsA versus CsA (p)			0.001			0.000	0.000	0.000
AS + CsA versus AS (p)			0.000			0.000	0.000	0.000
AS and CsA 48h viability	C1	A2.5	C1+A2.5	C5	A5	C5+A5	C1+A5	C5+A2.5
Plate 1	0.5586	0.8018	0.1317	0.2265	0.5124	0.0497	0.0722	0.0462
Plate 2	0.6622	0.8648	0.1701	0.2507	0.5612	0.0609	0.0969	0.0656
Plate 3	0.7520	0.9438	0.1786	0.2211	0.5440	0.0531	0.0800	0.0593
Plate 4	0.5713	0.7807	0.1444	0.2028	0.4882	0.0479	0.0760	0.0551
AS + CsA versus CsA (p)			0.00116			0.00000	0.00102	0.00000
AS + CsA versus AS (p)			0.00000			0.00000	0.00000	0.00000

Table S1. Statistical analysis of the differential viability after AS and CsA treatment alone or in combination for 24 or 48 hours in HCT116 cells (see Fig. 1A)

Characteristics	N=90 (%)
Age	
<70	44 (48.9)
≥70	46 (51.1)
Sex	
Male	47 (52.2)
Female	43 (47.8)
Tumor size (cm)	
≤5	46 (51.1)
＞5	42 (46.7)
Unknown	2 (2.2)
Histologic grade	
I	8 (8.9)
II	48 (53.3)
II-III	28 (31.1)
III	6 (6.7)
Lymph Node Involvement	
Negative	57 (63.3)
Positive	33 (36.7)
Stage	
I	7 (7.8)
II	47 (52.2)
III	32 (35.6)
IV	2 (2.2)
unknown	2 (2.2)

Table S2: Characteristics of 90 colon cancer cases with immunostaining

	Nuclear NFATc1 Negative N=44 (48.9%)	Positive N=46 (51.1%)	p
Age			
<70	20 (45.5)	24 (54.6)	0.524
≥70	24 (52.2)	22 (47.8)	
Sex			
Male	24 (51.1)	23 (48.9)	0.667
Female	20 (46.5)	23 (53.5)	
Tumor size (cm, n=88)			
≤5.0	22 (47.8)	24 (52.2)	0.839
>5.0	21 (50)	21 (50)	
Lymph node involvement			
Negative	31 (54.4)	26 (45.6)	0.170
Positive	13 (39.4)	20 (60.6)	
Grade			
I, I-II, II	32 (57.1)	24 (42.9)	0.044
II-III, III	12 (35.3)	22 (64.7)	
Stage (n=88)			
I and II	29 (53.7)	25 (46.3)	0.252
III and IV	14 (41.2)	20 (58.8)	
Median survival (95% CI)			
	69.8 (60.2-79.5)	53.2 (42.9-63.6)	0.041

Table S3: Characteristics of cases with negative or positive nuclear NFATc1 expression

	NFATc2 expression			NFATc2-cytoplasmic expression		
	Negative	Positive		Negative	Positive	
	N=18 (20%)	N=72 (80%)	p	N=21 (23.3%)	N=69 (76.7%)	p
Age						
<70	7 (15.9)	37 (84.1)	0.343	10 (22.7)	34 (77.3)	0.894
≥70	11 (23.9)	35 (76.1)		11 (23.9)	35 (76.1)	
Sex						
Male	10 (21.3)	37 (78.7)	0.752	11 (23.4)	36 (76.6)	0.987
Female	8 (18.6)	35 (81.4)		10 (23.3)	33 (76.7)	
Tumor size (cm, n=88)						
≤5.0	12 (26.1)	34 (73.9)	0.170	13 (28.26)	33 (71.7)	0.311
>5.0	6 (14.3)	36 (85.7)		8 (19.05)	34 (80.9)	
Lymph node involvement						
Negative	10 (17.5)	47 (82.5)	0.444	11(19.3)	46 (80.7)	0.234
Positive	8 (24.2)	25 (75.8)		10 (30.3)	23 (69.7)	
Grade						
I-II	13 (23.2)	43 (76.8)	0.328	13 (23.2)	41 (76.8)	0.704
III	5 (14.7)	29 (85.3)		7 (20.6)	27 (79.4)	
Stage (n=88)						
I and II	10 (18.5)	44 (81.5)	0.570	11(20.4)	43 (79.6)	0.333
III and IV	8 (23.5)	26 (76.5)		10 (29.4)	24 (70.6)	
Median survival						
	49.4 (35.5-63.4)	64.5 (56.2-72.8)	0.048	44.8 (31.7-57.8)	66.6 (58.2-74.9)	0.005

Table S4: Characteristics of cases with positive or negative total or cytoplasmic only NFATc2 expression.

	Univariate analysis			Multivariate analysis		
Covariant	HR	95% CI	P	HR	95% CI	P
Age	1.887	1.055-3.376	0.031	2.024	1.125-3.641	0.018
Stage	2.533	1.438-4.459	0.001	2.510	1.414-4.456	0.002
NFATc1-nuclear	1.838	1.037-3.257	0.041	1.931	1.082-3.445	0.030
NFATc2-cytoplasm	0.456	0.253-0.823	0.009	0.535	0.295-0.970	0.041
Sex	0.782	0.444-1.379	0.415			
Grade	1.436	0.812-2.541	0.220			
NFATc1-cytoplasm	0.699	0.385-1.270	0.224			
NFATc2-nuclear	1.284	0.706-2.336	0.428			

Table S5. Univariate and multivariate analysis of overall survival by the impact of age, stage, sex, grade, NFATc1 and c2 expression. Univariate and multivariate analysis of age, stage, NFATc1 nuclear expression, NFATc2-cytoplasmic expression, sex, histologic grade, NFATc1 cytoplasmic expression and NFATc2 nuclear expression of 88 cases(excluding 2 cases with unkown stage information). After multivariate analysis, NFATc1 nuclear expression remains significantly associated with worse patient survival, while NFATc2 cytoplasmic expression remains significantly associated with better survival.
HR, hazardous ratio. CI, confidence interval.
